# Sotatercept-related peripheral microvascular alterations in systemic sclerosis–associated pulmonary arterial hypertension: insights from nailfold capillaroscopy

**DOI:** 10.1093/rap/rkag124

**Published:** 2026-10-08

**Authors:** Yuichiro Segawa, Takehiro Takahashi, Jun Yamamoto, Kojiro Segawa, Takuya Takahashi, Moyuka Wada-Irimada, Yusuke Muto, Toshiya Takahashi, Nobuhiro Kikuchi, Saori Yamamoto, Haruka Sato, Nobuhiro Yaoita, Taijyu Satoh, Satoshi Yasuda, Yoshihide Asano

**Affiliations:** Department of Dermatology, Tohoku University Graduate School of Medicine, Sendai, Japan; Department of Dermatology, Tohoku University Graduate School of Medicine, Sendai, Japan; Healthspan Research Center (HeSReC), Tohoku University, Sendai, Japan; Department of Dermatology, Tohoku University Graduate School of Medicine, Sendai, Japan; Department of Dermatology, Tohoku University Graduate School of Medicine, Sendai, Japan; Department of Dermatology, Tohoku University Graduate School of Medicine, Sendai, Japan; Department of Dermatology, Tohoku University Graduate School of Medicine, Sendai, Japan; Department of Dermatology, Tohoku University Graduate School of Medicine, Sendai, Japan; Department of Dermatology, Tohoku University Graduate School of Medicine, Sendai, Japan; Department of Cardiovascular Medicine, Tohoku University Graduate School of Medicine, Sendai, Japan; Department of Cardiovascular Medicine, Tohoku University Graduate School of Medicine, Sendai, Japan; Department of Cardiovascular Medicine, Tohoku University Graduate School of Medicine, Sendai, Japan; Department of Cardiovascular Medicine, Tohoku University Graduate School of Medicine, Sendai, Japan; Department of Cardiovascular Medicine, Tohoku University Graduate School of Medicine, Sendai, Japan; Department of Medical Science and Innovation, SiRIUS Institute of Medical Research, Tohoku University, Sendai, Japan; Healthspan Research Center (HeSReC), Tohoku University, Sendai, Japan; Department of Cardiovascular Medicine, Tohoku University Graduate School of Medicine, Sendai, Japan; Department of Dermatology, Tohoku University Graduate School of Medicine, Sendai, Japan; Healthspan Research Center (HeSReC), Tohoku University, Sendai, Japan

*Rheumatology* Key message

- Sotatercept improves PAH while inducing structural alterations in peripheral microvasculature, causing dilation and micro-haemorrhages.

Dear Editor, SSc is a systemic autoimmune disease characterized by vasculopathy and fibrosis of the skin and internal organs [1]. CTD–associated pulmonary arterial hypertension (CTD-PAH) is a severe form of pulmonary hypertension, most commonly associated with SSc [2]. SSc–associated PAH (SSc-PAH) is associated with particularly poor survival, with a 5-year survival rate of ∼42% [3], partly reflecting progressive endothelial injury, vascular remodelling and fibrosis [4]. Sotatercept is an activin signalling inhibitor recently approved for PAH by the FDA in 2024. While sotatercept has been shown to restore pulmonary vascular homeostasis and has demonstrated efficacy in PAH, adverse events such as telangiectasia and bleeding have also been reported [5], raising the possibility of systemic microvascular alterations. However, peripheral microvascular changes during sotatercept treatment remain unclear. Nailfold videocapillaroscopy (NVC) enables noninvasive longitudinal assessment of microvascular abnormalities in SSc. Here, we report characteristic dynamics of microvascular structures observed with NVC in a patient with SSc-PAH treated with sotatercept.

A 70-year-old woman developed Raynaud’s phenomenon, exertional dyspnoea and skin sclerosis extending to the forearms 10 years earlier and was diagnosed with lcSSc. Antinuclear antibody was positive at 1:640 (discrete speckled pattern), and anti-centromere antibody was positive (196 U/ml). During follow-up, transthoracic echocardiography revealed an elevated tricuspid regurgitation pressure gradient (TRPG) of 34 mmHg, and she was referred to our hospital. Pulmonary function testing showed %VC of 83.6%, %FVC of 86.9% and %DLCO of 76.6%. High-resolution computed tomography (HRCT) showed only minimal reticular opacities at the lung bases, without clinically significant interstitial lung disease (ILD). Right heart catheterization demonstrated a mean pulmonary arterial pressure (mPAP) of 38 mmHg, a pulmonary arterial wedge pressure (PAWP) of 5 mmHg and a pulmonary vascular resistance (PVR) of 7.1 Wood units, confirming a pre-capillary haemodynamic profile consistent with SSc-PAH. PAH-specific therapy was initially started with tadalafil, which was discontinued because of severe neck flushing and replaced with riociguat. Macitentan was added but reduced because of eyelid oedema. Selexipag was subsequently added, temporarily discontinued because of lower-extremity pain and later restarted at a reduced dose. At sotatercept initiation, the patient was receiving riociguat 6 mg/day, macitentan 5 mg/day and selexipag 0.2 mg/day. She also received limaprost alfadex for Raynaud’s phenomenon, without antiplatelet or anticoagulant therapy. No treatment changes were made during the observation period. PAH-specific therapy reduced mPAP to 22 mmHg; however, further dose escalation was limited by adverse effects. Therefore, sotatercept was introduced for further therapeutic intensification. Three months after the initiation of sotatercept, mPAP and PVR were ameliorated (Fig. 1A). PAWP remained unchanged, supporting a pre-capillary haemodynamic profile (Fig. 1A). Although cardiac index (CI) decreased from 4.34 to 3.47 l/min/m^2^, it remained within the normal range, likely reflecting normalization of a previously hyperdynamic circulatory state rather than deterioration of right ventricular function (Fig. 1A).

**Figure 1:**
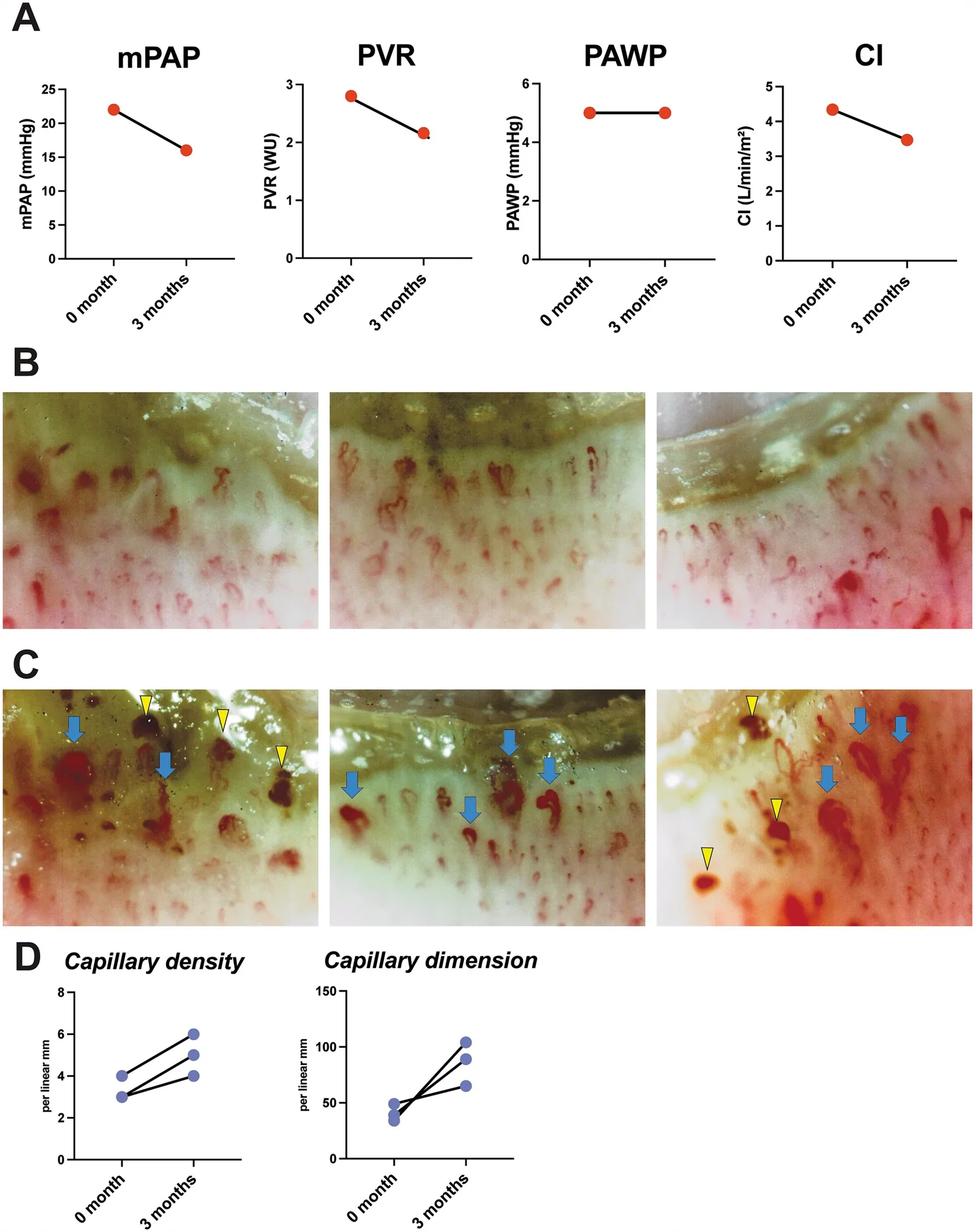
PAH parameters and NVC findings before and 3 months after sotatercept. (A) Right heart catheterization parameters before and 3 months after sotatercept: mPAP, PVR, PAWP and CI. (B) NVC images before treatment (×200). The central nailfold of the left fourth digit was assessed using three consecutive images under a standardized protocol [6], at 20–22°C after 15 min of acclimatization. (C) NVC images 3 months after treatment. Dilated abnormal capillaries (solid arrows) and micro-haemorrhages (arrowhead). (D) Capillary density and diameter before and 3 months after treatment were assessed from three images. Density was defined as distal-row capillaries per linear millimetre, and diameter as the apical diameter of the largest capillary

NVC was performed before and 3 months after sotatercept initiation, in November and February, respectively. Before treatment, capillary density and diameter were reduced, consistent with late-phase SSc vascular changes [6] (Fig. 1B). Across the three fields, capillary density increased from 3, 3 and 4 to 4, 5 and 6 capillaries/mm, and capillary dimension from 49, 34 and 39 to 65, 104 and 89 μm, respectively (Fig. 1B–D). Some newly developed capillaries had abnormal dilatation and irregular morphology. Some appeared ruptured, and multiple micro-haemorrhages were observed (Fig. 1C). Importantly, around this time, the patient began to report frequent episodes of gingival bleeding and epistaxis.

Alterations in bone morphogenetic protein/transforming growth factor-β (BMP/TGF-β) family signalling, including activin signalling, contribute to PAH pathogenesis [7]. Sotatercept is a fusion protein comprising the extracellular domain of ACTRIIA linked to human IgG1 Fc and acts as a ligand trap for several TGF-β superfamily members, including activins, BMP-9 and BMP-10 [5]. By sequestering activins, sotatercept suppresses abnormal vascular smooth muscle cell proliferation and pathological vascular remodelling [5]. A recent proteomics study showed that sotatercept reduces circulating BMP-9 and BMP-10 levels and downregulates proteins involved in cell–cell adhesion and vascular stability, including cadherin-2 (CDH2) and thrombospondin-2 (THBS2) [7]. Notably, combined BMP-9/BMP-10 deficiency in rodents protects against PAH but induces a vascular phenotype resembling hereditary haemorrhagic telangiectasia (HHT) [8]. The NVC changes were temporally associated with sotatercept initiation and new gingival bleeding and epistaxis, with no changes in concomitant vasoactive therapies. However, SSc progression and seasonal variability cannot be excluded, and causality cannot be established. Assessment of a single digit and measurement solely in the largest capillary in each field may limit representativeness. Any resemblance to HHT-related vascular fragility remains speculative.

In summary, pulmonary haemodynamic ameliorated after sotatercept treatment in a patient with SSc-PAH, while NVC demonstrated pronounced capillary dilation and increased micro-haemorrhages. These findings raise the possibility that sotatercept may be associated with peripheral microvascular changes reminiscent of the vascular fragility observed in HHT. Whether such changes may affect the underlying vasculopathy of SSc warrants prospective investigation.

## Data Availability

The data underlying this article will be shared on reasonable request to the corresponding author by qualified investigators engaged in independent scientific research.
